# Multi-Targeted Molecular Docking, Pharmacokinetics, and Drug-Likeness Evaluation of Okra-Derived Ligand Abscisic Acid Targeting Signaling Proteins Involved in the Development of Diabetes

**DOI:** 10.3390/molecules26195957

**Published:** 2021-10-01

**Authors:** Syed Amir Ashraf, Abd Elmoneim O. Elkhalifa, Khalid Mehmood, Mohd Adnan, Mushtaq Ahmad Khan, Nagat Elzein Eltoum, Anuja Krishnan, Mirza Sarwar Baig

**Affiliations:** 1 Department of Clinical Nutrition, College of Applied Medical Sciences, University of Hail, Hail 2440, Saudi Arabia; ao.abdalla@uoh.edu.sa (A.E.O.E.); nagacademic0509@gmail.com (N.E.E.); 2 Department of Pharmaceutics, College of Pharmacy, University of Hail, Hail 2440, Saudi Arabia; adckhalid@gmail.com; 3 Department of Biology, College of Science, University of Hail, Hail 2440, Saudi Arabia; drmohdadnan@gmail.com; 4 Department of Microbiology and Immunology, College of Medicine and Health Sciences, UAE University, Al Ain 15551, United Arab Emirates; mushtaq.khan@uaeu.ac.ae; 5 Department of Molecular Medicine, School of Interdisciplinary Sciences & Technology, Jamia Hamdard, New Delhi 110062, India; anuja.krishnan@jamiahamdard.ac.in

**Keywords:** anti-diabetic, okra, abscisic acid, nutraceuticals, *Diabetes mellitus*, molecular docking, phytohormones

## Abstract

*Diabetes mellitus* is a global threat affecting millions of people of different age groups. In recent years, the development of naturally derived anti-diabetic agents has gained popularity. Okra is a common vegetable containing important bioactive components such as abscisic acid (ABA). ABA, a phytohormone, has been shown to elicit potent anti-diabetic effects in mouse models. Keeping its anti-diabetic potential in mind, in silico study was performed to explore its role in inhibiting proteins relevant to diabetes mellitus- 11β-hydroxysteroid dehydrogenase (11β-HSD1), aldose reductase, glucokinase, glutamine-fructose-6-phosphate amidotransferase (GFAT), peroxisome proliferator-activated receptor-gamma (PPAR-gamma), and Sirtuin family of NAD(+)-dependent protein deacetylases 6 (SIRT6). A comparative study of the ABA-protein docked complex with already known inhibitors of these proteins relevant to diabetes was compared to explore the inhibitory potential. Calculation of molecular binding energy (ΔG), inhibition constant (pKi), and prediction of pharmacokinetics and pharmacodynamics properties were performed. The molecular docking investigation of ABA with 11-HSD1, GFAT, PPAR-gamma, and SIRT6 revealed considerably low binding energy (ΔG from −8.1 to −7.3 Kcal/mol) and predicted inhibition constant (pKi from 6.01 to 5.21 µM). The ADMET study revealed that ABA is a promising drug candidate without any hazardous effect following all current drug-likeness guidelines such as Lipinski, Ghose, Veber, Egan, and Muegge.

## 1. Introduction

Diabetes is one of the most prevalent epidemics, affecting almost 382 million people worldwide. According to the International Diabetes Federation (IDF) report, it is alleged that approximately 1.3 million people die from diabetes every year. IDF suggests that around 629 million people will have diabetes by 2045 worldwide [1].

Diabetes is a chronic metabolic disorder characterized by insulin resistance and pancreatic β-cell dysfunction caused by uncontrolled hyperglycemia. Altered sugar, fat, and protein metabolism in diabetes and associated complications include retinopathy, neuropathy, nephropathy, cardiovascular diseases, skin complications, and macrovascular complications [2]. This represents a major economic burden since 12% of global health expenditure is spent on the diabetic population. Type 2 diabetes mellitus (T2DM) is a complex disease characterized by high glucose plasma levels due to insufficient insulin secretion or action or both affecting people of all age groups [2,3]. Additionally, insulin resistance in target tissues and a relative deficiency of insulin secretion from pancreatic β-cells are the major metabolic issue with diabetes. In response to nutrient spillover in insulin resistance and eventual β-cell dysfunction, the general fuel homeostasis of the body gets altered. β-cell hyperplasia and hyperinsulinemia in response to insulin resistance occur in the preclinical period of the disease.

As a consequence of the failure of β-cells to compensate for insulin resistance, relative insulin deficiency progresses into diabetes [4]. Diabetes involves various cellular pathways such as insulin secretion, insulin resistance, and carbohydrate absorption. Some human proteins have been identified as key regulators in the development of diabetes like glucokinase, AMP-activated protein kinase, 11 β-hydroxysteroid dehydrogenases (11 β-HSD), insulin receptor substrate, interleukin1 beta, dipeptidyl peptidase IV, glutamine-fructose-6-phosphate amidotransferase (GFAT), peroxisome proliferator-activated receptor-gamma (PPAR-gamma), tyrosine phosphatases, tyrosine kinase insulin receptor, protein kinase B, and insulin receptor [3]. Various therapeutic interventions have been developed to treat and manage diabetes, including dietary modifications, exercise, and anti-diabetic agents. However, reports suggest that anti-diabetic agents are usually associated with severe side effects or adverse effects, and sometimes, their efficacies are controversial. Hence, attention has been shifted towards traditional and alternative medicines or food-derived products rich in anti-diabetic phytoconstituents. Bioactive components present in plants and plant-derived products such as alkaloids, flavonoids, glycosides, gum, carbohydrates, triterpenes, and verities of short-peptides are usually responsible for their therapeutic importance [5].

Okra has recently been recognized for its potential therapeutic purposes because of various important phytochemical constituents. Okra plant [*Abelmoschus esculentus* (L.) Moench] is a nutritive vegetable known by various names such as lady’s finger, green ginseng, and plant Viagra [6]. Presently, okra is used for its nutritional values and nutraceutical and therapeutic properties, owing to various important bioactive compounds and their associated therapeutic properties [7].

**Figure 1:**
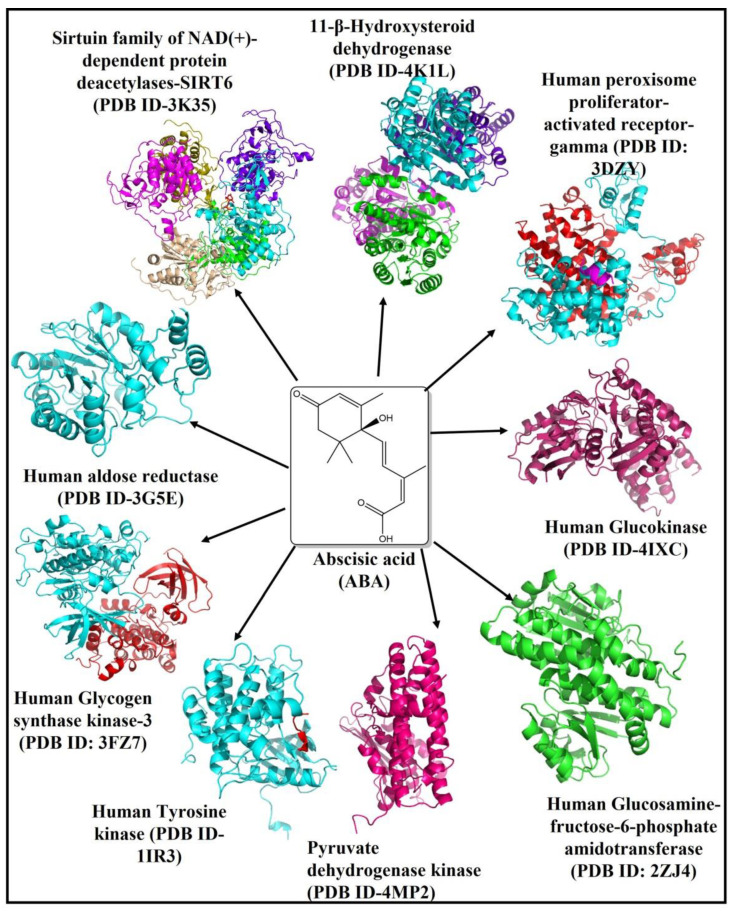
The chemical structure of ABA (center). The human proteins involved in the development of diabetes are 11β-Hydroxysteroid dehydrogenase [11β-HSD (PDB ID-4K1L)], aldose reductase (PDB ID-3G5E), glucokinase (PDB ID-4IXC), glycogen synthase kinase-3 (PDB ID-3F7Z), glucosamine-fructose-6-phosphate amidotransferase [GFAT (PDB ID-2ZJ4)], pyruvate dehydrogenase kinase (PDB ID-4MP2), peroxisome proliferator-activated receptor-gamma (PDB ID-3DZY), Sirtuin family of NAD(+)-dependent protein deacetylases [SIRT6 (PDB ID-3K35)] and Tyrosine kinase (PDB ID-1IR3), were individually docked with ABA.

The profile of the bioactive components present in different parts of okra has been reported, which includes polyphenolic compounds, flavanol derivatives, carotene, protein (i.e., high lysine levels), folic acid, thiamine, riboflavin, niacin, vitamin C, oxalic acid, amino acids, oligomeric catechins, and newly identified bioactive component abscisic acid [8]. ABA (MW = C15H20O4, IUPAC = (2Z,4E)-5-[(1S)-1-hydroxy-2,6,6-trimethyl-4-oxocyclohex-2-en-1-yl]-3-methylpenta-2,4-dienoic acid), shown in Figure 1, is a compound naturally present in fruits and vegetables. Its concentration varies depending on the type of vegetables ranging from 0.29 mg/kg to 0.62 mg/kg of the wet weight of vegetables and fruits, respectively. Throughout the life cycle of plants, it is involved in various physiological and developmental activities, where it regulates seed maturation, maintenance of embryo dormancy and plays a relevant role in various processes against environmental stressors [9]. In recent years, ABA was found to be associated with human diseases, and is currently being investigated for various therapeutic purposes such as diabetes, prostate cancer, Alzheimer’s, and other neurodegenerative diseases, due to broad biological activity spectrum of ABA [10,11]. Mechanisms of action underlying the observed anti-diabetic effects are insulin secretion, insulin resistance, and carbohydrate absorption. The search for new therapeutic targets remains a challenge; the present work investigates the anti-diabetic potential of ABA of okra in silico approach by predicting the binding interactions between ABA with target proteins involved in the development of diabetes mellitus.

## 2. Results & Discussion

Molecular docking and virtual screening are fast, economical, and reliable approaches for identifying both a potential druggable protein target as well as a novel drug (lead molecule) through rational drug designing (RDD) or computer-aided drug design (CADD). RDD or CADD is now being used to annotate and evaluate large pharmacological libraries swiftly.

This study applies molecular docking-based virtual screening to identify a promising target for T2DM. Based on literature review and available crystal structures of proteins involved in several biosynthetic pathways as a key regulator in T2DM, we selected nine human proteins 11β-hydroxysteroid dehydrogenase [11β-HSD (PDB ID-4K1L)], aldose reductase (PDB ID-3G5E), glucokinase (PDB ID-4IXC), glycogen synthase kinase-3 [GSK-3 (PDB ID-3F7Z)], glucosamine:fructose-6-phosphate amidotransferase [GFAT (PDB ID-2ZJ4)], pyruvate dehydrogenase kinase (PDB ID-4MP2), peroxisome proliferator-activated receptor-gamma (PDB ID-3DZY), sirtuin family of NAD(+)-dependent protein deacetylases [SIRT6 (PDB ID-3K35)] and Tyrosine kinase (PDB ID-1IR3), shown in Figure 1. The plausible molecular/atomic interactions of ABA with these proteins were investigated in this in silico study. 

ABA was found to have a binding energy of −8.1 kcal/mol, −7.3 kcal/mol, −7.3 kcal/mol, −7.3 kcal/mol, −6.8 kcal/mol, −6.6 kcal/mol, −6.6 kcal/mol, 6.3 kcal/mol, and −6.2 kcal/mol, with 11β-HSD1, GFAT, PPAR-gamma, SIRT6, glucokinase, aldose reductase, glycogen synthase kinase-3, Pyruvate dehydrogenase kinase (PDK), and Tyrosine kinase proteins, respectively (Table 1). The binding energy (Kcal/mol) would be used to correlate and investigate the binding affinity of various ligands or inhibitors with their corresponding protein target. In general, the lower the binding energy, the greater the ligand’s affinity for the receptor protein will be. As a result, the ligand with the highest affinity can be taken forward as a candidate for further research.

### 2.1. Abscisic Acid Is a Potent Inhibitor of the Human 11β-Hydroxysteroid Dehydrogenase Type 1 (11β-HSD1) Enzyme 

Intracellular conversion of metabolically inert cortisone to active cortisol using NADPH as a co-factor is carried out by the 11β-HSD1 enzyme [12,13,14,15]. Cortisol increases hepatic glucose production by inducing genes involved in gluconeogenesis and glycogenolysis in the liver. Cortisol promotes pre-adipocyte differentiation into mature adipocytes, resulting in adipose tissue hyperplasia. By modulating cortisone/cortisol levels, selective inhibition of this enzyme can be a novel treatment for T2DM and hyperlipidemia [16,17,18,19]. Obesity, diabetes, wound healing, and muscular atrophy are glucocorticoid-related disorders, and inhibiting 11β-HSD1 has many therapeutic values, including T2DM and hyperlipidemia.

The X-ray crystallography investigations of the crystal structure of an inhibitor molecule (4aS,8aR)-3-(cyclohexylamino)-4a,5,6,7,8,8a-hexahydrobenzo[e][1,3,4]oxathiazine 1,1-dioxide (C13H22N2O3S) docked with human and murine 11β-HSD1 proteins revealed that its cyclohexyl-NH interacts with the key active site residue Tyr183 [17]. Furthermore, one of the sulfonyl oxygen atoms forms a hydrogen bond to the main-chain nitrogen of Ala172 of 11β-HSD1 protein. Meanwhile, the side chain Tyr177 of the human 11β-HSD1 enzyme typically forms Van der Waals interaction to the same inhibitor molecule [17]. 

**Figure 2:**
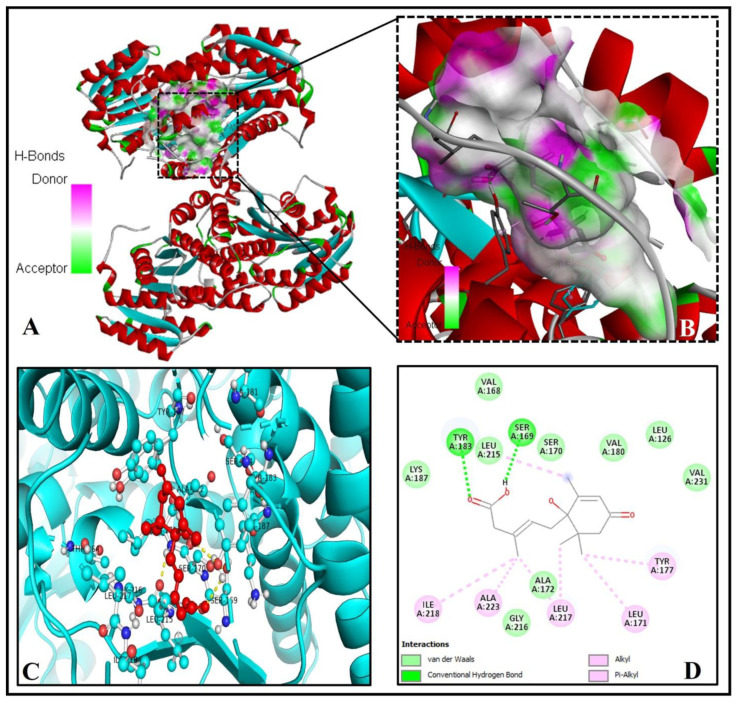
Significant molecular bonding of ABA with 11β-hydroxysteroid dehydrogenase1 [11β-HSD1 (PDB ID- 4K1L). (**A**) The coupling of ABA with active center residues. (**B**) The enlarged image shows the hydrogen bonds donor and acceptor amino acid residues in the junction cavity. (**C**) The 3D image shows a significant interaction of ABA (red ball and sticks) with functionally important residues (cyan ball and sticks) of human 11β-HSD1. (**D**) The 2D plot is showing the interaction of the binding pocket residues with the ABA inhibitor.

Interestingly in our in silico analysis, we found that key residues of active site viz., TYR(A)183 and SER(A)169 of the human 11β-HSD1 enzyme interact with the two oxygen atoms of the 3-Methylpenta-2,4-dienoic acid substructure of abscisic acid [(2Z,4E)-5-[(1S)-1-hydroxy-2,6,6-trimethyl-4-oxocyclohex-2-en-1-yl]-3-methylpenta-2,4-dienoic acid)]. Nine other amino acid residues of the human 11β-HSD1 enzyme, LEU(A)126, VAL(A)168, SER(A)170, ALA(A)172, VAL(A)180, LYS(A)187, LEU(A)215, GLY(A)216, and VAL(A)231 formed Van-der-Waals interaction with ABA (Figure 2A–D). This interaction showed the lowest binding energy of −8.1 kcal/mol and the highest inhibition constant of 6.01 µM, respectively (Table 1). The pi-pi stacking interaction by LEU171, TYR177, LEU217, ILE218, and ALA223 with other pi-cation and pi-alkyl interactions may help in stabilizing the ABA bounded with the active residues of the 11β-HSD1 enzyme (Figure 2D). Based on these very similar binding patterns and docking complex analysis, we can say that ABA is the true, potent inhibitor of the human 11β-HSD1 enzyme, thus possibly help in controlling T2DM and hyperlipidemia.

### 2.2. Abscisic Acid Significantly Binds and Inhibits Glutamine: Fructose-6-Phosphate Amidotransferase (GFAT) Effectively

Glutamine-fructose-6-phosphate amidotransferase (GFAT) is a rate-limiting enzyme in the hexosamine biosynthetic pathway a key regulator in T2DM [20,21,22]. In mammals, glucose integration via the hexosamine biosynthetic pathway is regarded as a cellular nutrition sensor. This pathway is one of the strategies by which hyperglycemia induces peripheral insulin resistance [23,24]. In vitro and in vivo studies indicate the association of hyperactivity of human GFAT with insulin resistance, thus qualifying it as a promising candidate for T2DM [25].

The C-terminal 40 kDa isomerase domain of GFAT (residues Gln313–Glu680) contains the active site (near the CF helix) and is involved in converting fructose-6-phosphate (Fru6P) to glucosamine-6-phosphate (GlcN6P) utilizing ammonia (NH3) as a substrate. The X-ray diffraction pattern shows that the bound ligand AGP (2-deoxy-2-amino glucitol-6-phosphate) with GFAT (PDB ID 2ZJ4) establishes hydrogen bonds with Thr375, Ser376, Ser420, Gln421, Ser422, and Thr425 directly, and other hydrogen-bonds with Val471, His576, and Ala674 from the neighboring subunit [20].

**Figure 3:**
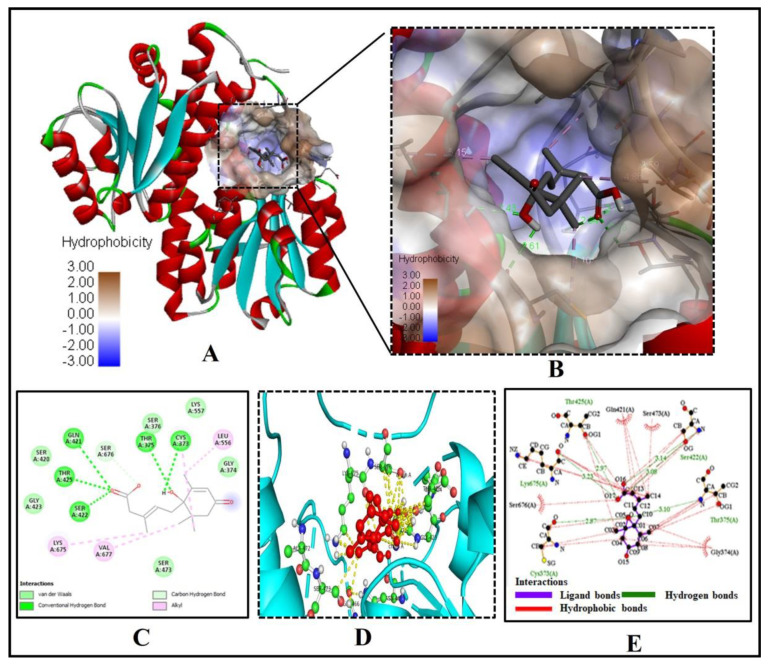
ABAs significant molecular binding with human glutamine-fructose-6-phosphate amidotransferase [GFAT (PDB ID- 2ZJ4)]. (**A**) ABA binding with the binding pocket. (**B**) Enlarged view showing the hydrophobicity of amino acid residues of the binding cavity (**C**) The 2D plot shows the molecular level interactions of binding pocket residues with the inhibitor ABA. (**D**) The 3D image shows strong interactions (yellow dashed lines) of ABA (red ball and sticks) with the functionally important residues (green ball and sticks) of human glutamine-fructose-6-phosphate amidotransferase. (**E**) The 2D LigPlot+ image shows the atomic level interactions of binding pocket residues with the ABA.

We found a similar interaction pattern of ABA with the active site amino acid residues of GFAT (PDB ID- 2ZJ4), suggesting ABA can potentially inhibit GFAT activity. Five key amino acid residues of GFAT viz., CYS373, THR375, GLN421, SER422, and THR425 were strongly forming hydrogen bonds of bond length 2.87Å, 3.10Å, 3.08Å, 3.14Å and 2.94 Å, respectively, suggesting a strong binding to the active pocket (Figure 3A–E). 

The three residues GLN421, SER422, and THR425 of the GFAT enzyme interact with two oxygen atoms of the 3-Methylpenta-2,4-dienoic acid substructure of ABA, while the remaining two residues CYS373, THR375 were forming a hydrogen bond with the oxygen atom of the 1-hydroxy-2,6,6-trimethyl-4-oxocyclohex-2-en-1-yl ring of ABA (Figure 3C). Similarly, seven residues of GFAT protein, GLY(A)374, SER(A)376, SER(A)420, GLY(A)423, SER(A)473, LYS(A)557 and SER(A)676, were forming Van der Waals interaction with ABA (Figure 3C–E). The low binding energy of −7.3 kcal/mol and inhibition constant of 5.21 µM (Table 1), and three alkyl interactions by LEU556, LYS675 and VAL677 residues with (1S)-1-hydroxy-2,6,6-trimethyl-4-oxocyclohex-2-en-1-yl ring helps in stabilizing the ABA bound with the active site. As a result, we may conclude that ABA is an effective inhibitor of the GFAT enzyme.

### 2.3. Binding of Abscisic Acid to the Catalytic Pocket of the Human Peroxisome Proliferator-Activated Receptor-Gamma (PPAR-Gamma)

PPAR-gamma is a major transcriptional factor (TF) that regulates adipogenesis, insulin sensitivity, and glucose homeostasis in humans [26,27]. The drug rosiglitazone, which acts as a ligand of PPAR-gamma, is an excellent insulin sensitizer, improving glucose absorption and lowering hyperglycemia and hyperinsulinemia [28,29,30]. 

The decreased ability of PPAR-gamma to bind DNA in response to rosiglitazone manifested the receptors’ inability to activate transcription. The PPARs are also potential therapeutic targets that could treat atherosclerosis, inflammation, and hypertension. Studies showed that in the crystal structures of PPAR-gamma and rosiglitazone complex, binding pockets of the intact PPAR-gamma receptor interact with the rosiglitazone, especially with the Gln193, Tyr189, Leu196, Ala197, Tyr192, Glu203, Lys201, Arg202, Asp166, Lys336, Asn335, Asp337, Leu237, Phe347, Val248, Glu351, and Tyr250 residues [30]. 

**Figure 4:**
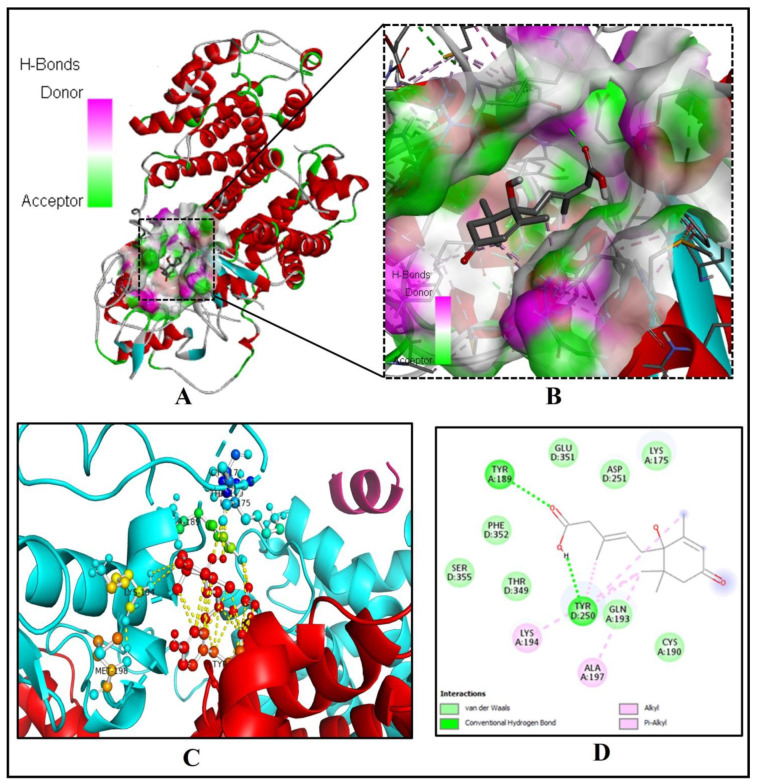
ABA docked with human peroxisome proliferator-activated receptor-gamma [PPAR-gamma (PDB ID: 3DZY)]. (**A**) The binding pocket residues of PPAR-gamma bind to ABA. (**B**) The binding cavity’s hydrogen bond donor and acceptor amino acid residues are shown in a magnified view. (**C**) The 3D image shows significant interactions (yellow dashed lines) of ABA (red ball and sticks) with the residues of the human PPAR-gamma (green, yellow, cyan ball and sticks). (**D**) The molecular level interactions of binding-pocket residues with the ABA are depicted in the 2D plot.

Our investigation discovered that crucial binding pocket residues of the human PPAR-gamma, TYR(A)189 and TYR(D)250, form two hydrogen bonds with the two oxygen atoms of the 3-Methylpenta-2,4-dienoic acid substructure of ABA (Figure 4A–D). 

The low binding energy of −7.3 kcal/mol and inhibition constant of 5.21 µM (Table 1) and two alkyl-pi-alkyl interactions by LYS194 and ALA197 with 1-hydroxy-2,6,6-trimethyl-4-oxocyclohex-2-en-1-yl ring of ABA may stabilize the ABA bounded with the active site (Figure 4D). Interestingly, eight residues of PPAR-gamma LYS(A)175, CYS(A)190, GLN(A)193, ASP(D)251, THR(D)349, GLU(D)351, PHE(D)352, and SER(D)355, were interacting via Van der Waals forces with ABA (Figure 4C,D).

Based on a similar binding and interaction pattern, we may conclude that ABA also acts as an inhibitor of PPAR-gamma, improving glucose absorption and lowering hyperglycemia.

### 2.4. The Binding Pattern of ABA with Human Mono-ADP-Ribosyl Transferase Sirtuin-6 (SIRT6)

SIRT6 is a prominent mammalian sirtuin (SIRT1–7) involved in various cellular processes such as glucose homeostasis maintenance, DNA repair, and longevity [31,32,33]. SIRT6 is NAD+- dependent deacetylases that target various acetylated proteins in mammals to regulate their cellular activity [34]. 

The monomeric crystal structure (2.0 Å) of human SIRT6 in complex with ADP-ribose showed that THR55, ASP61, PHE62, TRP69, ASP81, GLN111, HIS131, TRP186, GLY212, THR213, SER214, ILE217, TYR255, and VAL256 residues surround the ADP-ribose by hydrogen bonding network [31]. 

**Figure 5:**
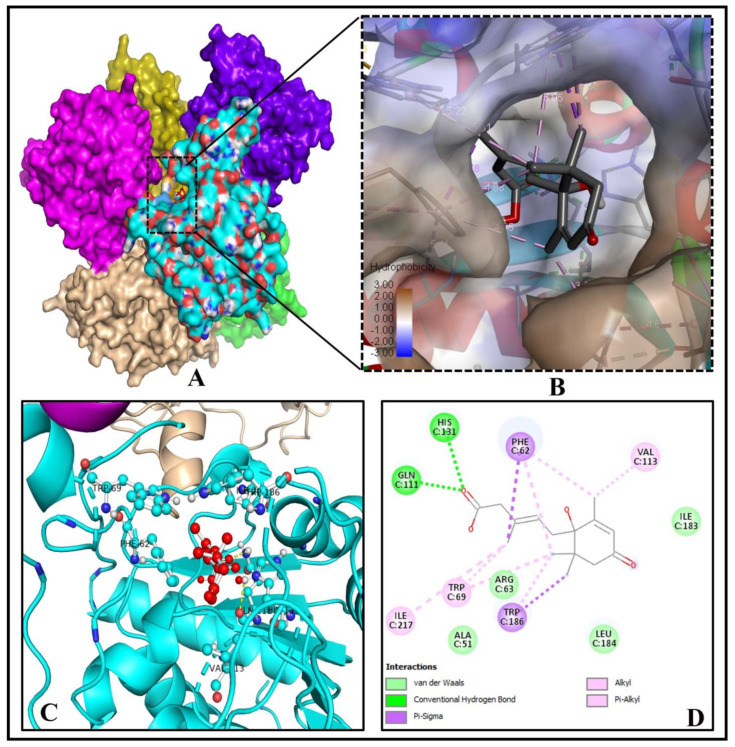
Molecular binding of ABA with human SIRT6 (PDB ID: 3K35). (**A**) ABA binds to the catalytic site. (**B**) Magnified view shows the hydrophobicity of amino acid residues of the binding cavity of SIRT 6 (**C**) 3D image showing interactions of ABA (red ball and sticks) with important residues of human SIRT6 (cyan ball and sticks). (**D**) 2D graph showing interactions at the molecular level pocket residues linked to the ABA.

Our study revealed that binding pocket residues (GLN111, HIS131 of chain C) of the hexameric human SIRT6 protein make two hydrogen bonds with the one oxygen atom of the 3-Methylpenta-2,4-dienoic acid (C_6_H_8_O_2_) substructure of ABA using molecular docking and pose prediction analysis (Figure 5A–D). Interestingly, four other residues of SIRT6 like ALA51, ARG63, ILE183 and LEU184 of chain C interact via Van der Waals forces with other atoms of ABA (Figure 5C,D). 

### 2.5. The Binding Pattern of ABA with Glucokinase

Glucokinase is the most abundant hexokinase in the liver, and it plays a critical role in blood glucose homeostasis because it has strong control over hepatic glucose disposal and serves as the glucose sensor for insulin secretion in pancreatic β-cells [35]. Glucokinase is currently regarded as a promising target of anti-hyperglycemic medicines to control T2DM, but this protein target’s mode of inhibition or activation is not fully understood.

There is a single published report on the crystal structure of human glucokinase (PDB ID- 4IXC) complexed with alpha-D-glucopyranose and (2S)-2-{[1-(3-chloropyridin-2-yl)-1H-pyrazolo[3,4-d]pyrimidin-4-yl]oxy}-*N*-(5-methylpyridin-2-yl)-3-(propan-2-yloxy) propanamide. These small molecules are regarded as activators of human glucokinase, but their exact mechanism of action and key amino residues involved in the interaction are not published yet.

**Figure 6:**
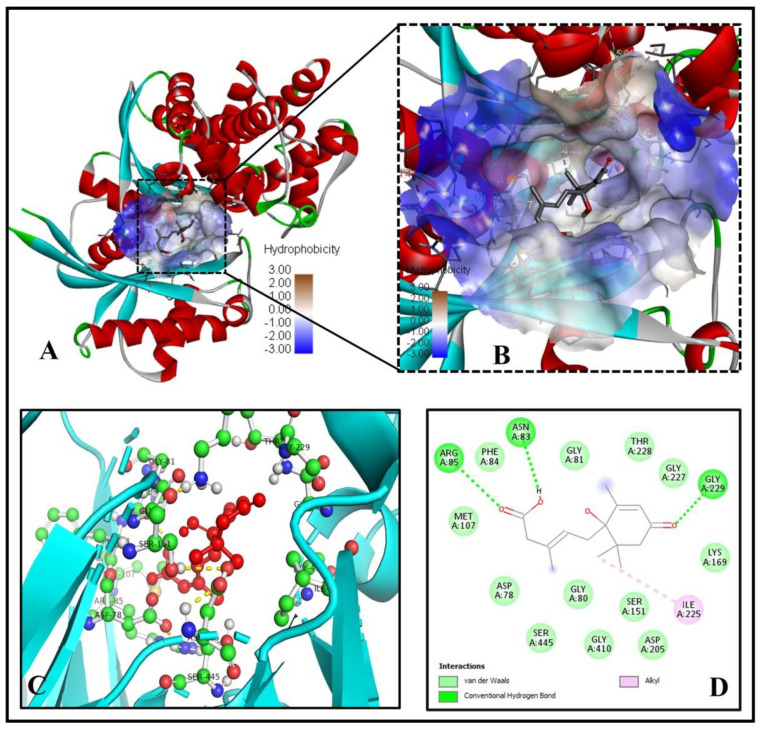
Human glucokinase (PDB ID- 4IXC) is docked with ABA. (**A**) ABA binds the catalytic site. (**B**) An enlarged image of the hydrophobicity of the binding cavity’s amino acid residues (**C**) The 3D picture reveals substantial interactions (yellow dashed lines with bond length) of ABA (red ball and sticks) with human glucokinase residues (green ball and sticks). (**D**) The molecular level interactions of binding pocket residues with ABA are depicted in the 2D plot.

Our docking and binding analysis exhibit a good binding pattern (binding energy = −6.8 Kcal/mol) of ABA with human glucokinase (PDB ID-4IXC) protein (Figure 6A,B), which is mediated through three hydrogen bonds forming residues ASN83, ARG85, and GLY229 and twelve via Van-der-Waals forces ASP78, GLY80, GLY81, PHE84, MET107, SER151, LYS169, ASP205, GLY227, THR228, GLY410, and SER445 (Figure 6C,D).

### 2.6. Analysis of the Molecular Binding Pattern of Abscisic Acid with Aldose Reductase

Aldose reductase is the rate-limiting enzyme in the polyol pathway. It converts excess D-glucose to D-sorbitol with NADPH as a co-factor [36]. It is crucial in the treatment of diabetic microvascular problems [37]. Aldose reductase is also involved in lipid metabolism.

**Figure 7:**
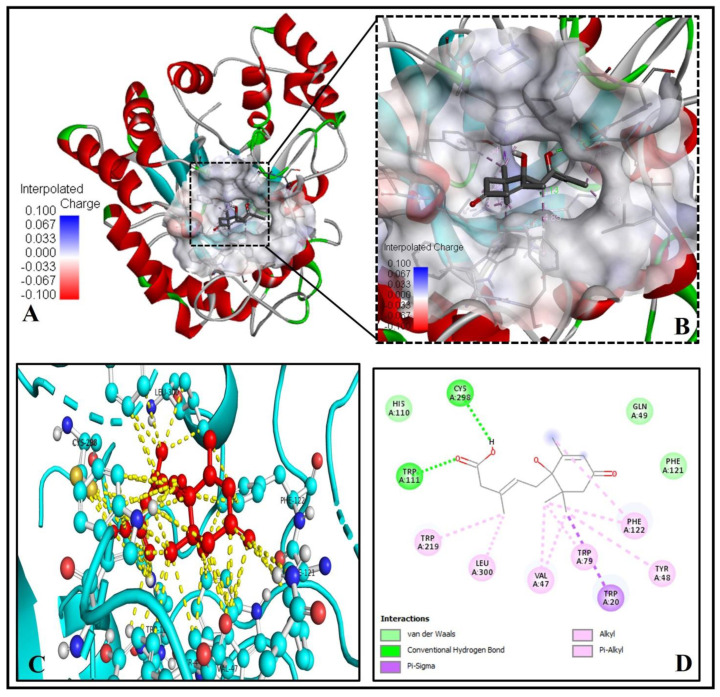
Molecular binding of ABA to aldose reductase (PDB ID: 3G5E). (**A**) Binding of ABA to the active site residues. (**B**) Magnified view showing the interpolated charge of amino acid residues of the binding cavity (**C**) The 3D image showing bonding (yellow dashed lines) of ABA (red ball and sticks) with residues of human aldose reductase (cyan ball and sticks). (**D**) 2D graph showing interactions at the molecular level of the binding compartment of aa residues with the ABA.

Our docking and binding analysis exhibit a good binding pattern (ΔG = −6.6 Kcal/mol) of ABA with aldose reductase (PDB ID- 3G5E) protein (Figure 7A–D), which is mediated through two hydrogen bonds forming residues TRP111, CYS298 and three via Van der Waals forces, GLU49, HIS110 and PHE121 (Figure 7C,D). Six alkyl-pi-alkyl interactions and one pi-pi sigma interaction by TRP20 with the 1-hydroxy-2,6,6-trimethyl-4-oxocyclohex-2-en-1-yl ring of ABA stabilize the ABA bounded with the active site of aldose reductase (Figure 7D).

### 2.7. Analysis of Abscisic Acid and Glycogen Synthase Kinase-3 (GSK-3) Docked Complex

GSK-3, a unique multifunctional serine/threonine kinase, is involved in the glycolysis pathway. GSK-3 is active and capable of synthesizing glycogen in its unphosphorylated state. PKB/AKT phosphorylates GSK-3 on serine 9 in response to insulin binding [38]. As a result, it is critical to the insulin signaling pathway’s transmission of regulatory and proliferative signals occurring at the cell membrane, potentially modulating blood glucose levels [38].

**Figure 8:**
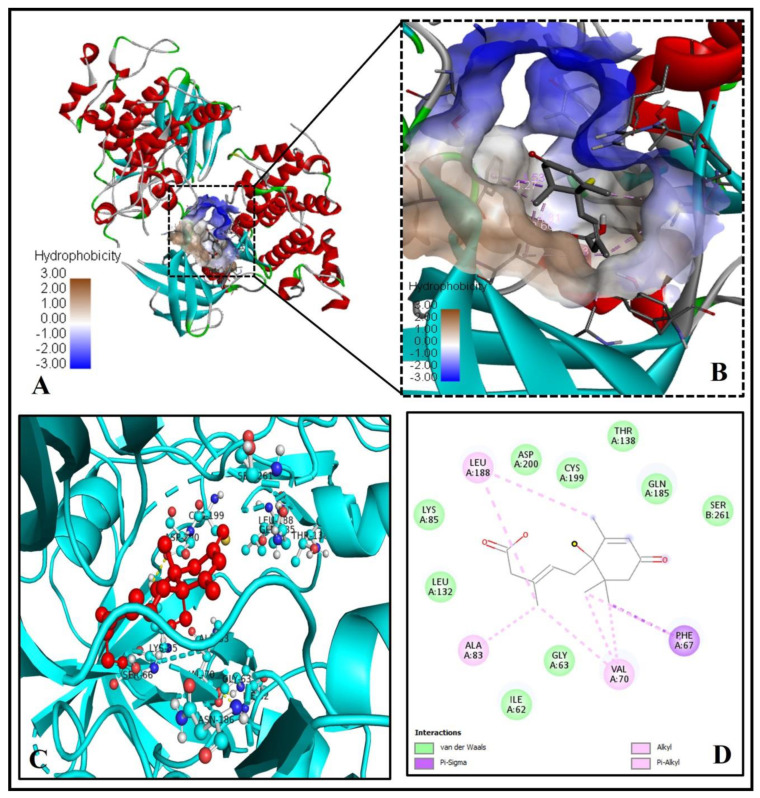
ABA shows mild interaction with human GSK3 (PDB ID: 3FZ7). (**A**) The ABA binds to the binding pocket near the catalytic center. (**B**) An enlarged image is showing the hydrophobicity of the binding cavity’s amino acid residues. (**C**) The 3D picture reveals substantial interactions (yellow dashed lines) of ABA (red ball and sticks) with human GSK-3 residues (cyan ball and sticks). (**D**) The molecular level interactions of binding pocket residues with the ABA are depicted in the 2D plot.

However, unlike previously mentioned proteins, GSK-3 (PDB ID -3F7Z) did not show a good binding pattern with ABA (binding energy = −6.6 Kcal/mol) (Figure 8A–D). There were no hydrogen bonds involved, only via Van der Waals forces, alkyl-pi-alkyl interaction, and pi-pi sigma interaction with ABA stabilizes the ABA bounded with the active site (Figure 8D).

### 2.8. Screening of Pyruvate Dehydrogenase Kinase (PKD) and Abscisic Acid Docked Complex

PKD negatively regulates the mitochondrial pyruvate dehydrogenase complex (PDC) activity by reversible phosphorylation. PDK isoforms are upregulated in obesity, diabetes, heart failure, and cancer and are potential therapeutic targets for these important human diseases [39].

**Figure 9:**
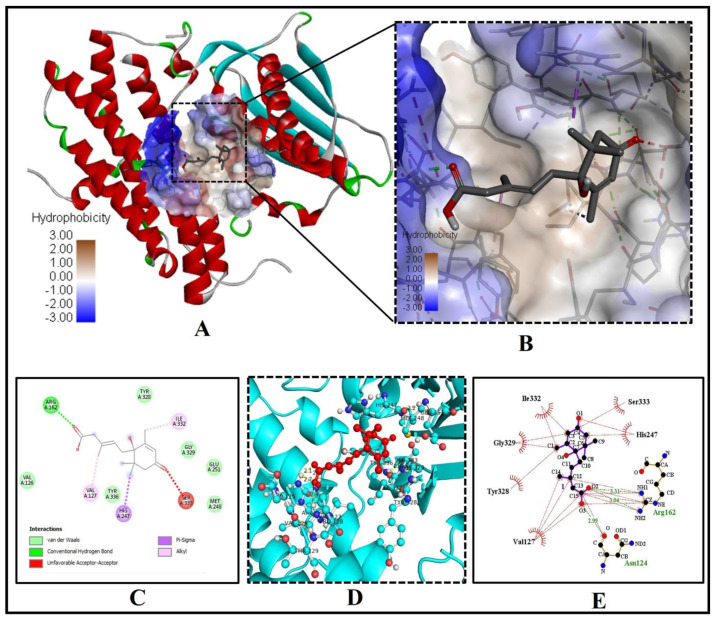
The docked complex of ABA with human pyruvate dehydrogenase kinase (PDB ID: 4MP2). (**A**) ABA binds with the catalytic site. (**B**) Magnified view showing the hydrophobicity of the amino acid fragments of the binding cavity (**C**) 2D graph showing the interactions at the molecular level of the bound cavity fragments with the inhibitor ABA. (**D**) The 3D image is showing interactions (with bond length) of ABA (red ball and sticks) with residues of human PKD (cyan ball and sticks). (**E**) 2D LigPlot is showing atomic level interactions of vesicle fragments of pyruvate dehydrogenase kinase bound to ABA.

Our analysis showed a poor binding pattern (binding energy = −6.3 Kcal/mol) of ABA with PKD (PDB ID- 4MP2) protein (Figure 9A–E) due to unfavorable repulsion. However, it forms hydrogen bonds, Van der Waals forces, alkyl-pi-alkyl interaction, and pi-pi sigma interaction with ABA (Figure 9C–E).

### 2.9. Investigation of the Docked Complex of Tyrosine Kinase with Abscisic Acid

**Figure 10:**
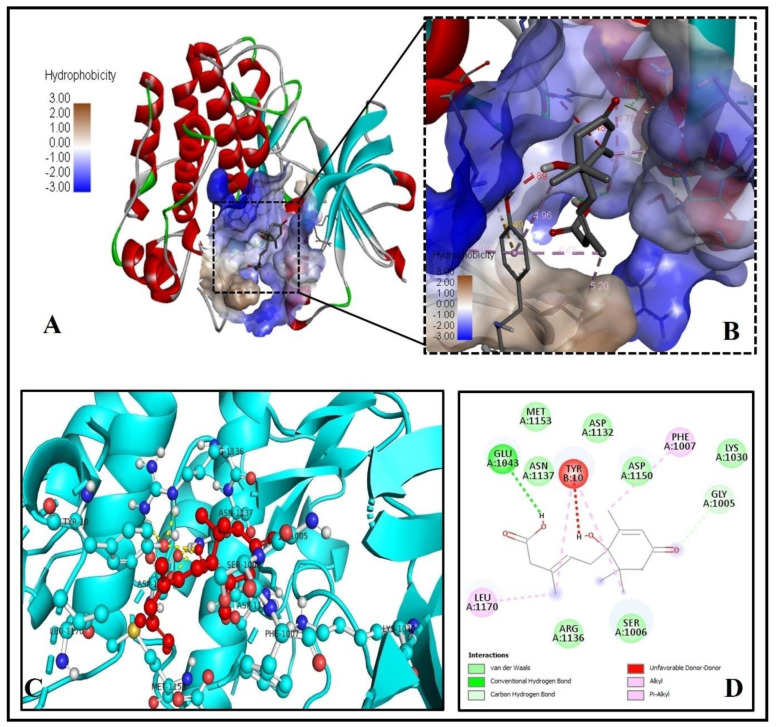
ABA molecular docking with human tyrosine kinase (PDB ID: 1IR3). (**A**) Binding of ABA to the active site of tyrosine kinase. (**B**) An enlarged image showing hydrophobicity of the binding cavity’s amino acid residues (**C**) The 3D picture depicts substantial interactions of ABA (red ball and sticks) with human tyrosine kinase’s essential residues (cyan ball and sticks) (**D**) The molecular level interactions of binding pocket residues with ABA are depicted in the 2D plot.

Further, ABA displayed a weak binding pattern (ΔG = −6.2 Kcal/mol) with the human tyrosine kinase (PDB ID-1IR3) protein (Figure 10A–D) due to unfavorable repulsion. However, it forms hydrogen bonds, Van der Waals forces, alkyl-pi-alkyl interaction, and pi-pi sigma interaction with ABA (Figure 10D).

### 2.10. Computational Pharmacodynamics Screening of Abscisic Acid Ligand

The molinspiration bioactivity score (v2018.03) is calculated and presented (Table 2) for active drug-likeness towards parameters like ion channel modulators, kinase inhibitors, GPCR ligands, nuclear receptor ligands, protease inhibitors, and other enzyme inhibitors with scores for >100,000 average drug-like molecules. The score allows efficient separation of active and inactive molecules. The higher values of bioactivity score of nuclear receptor ligand, enzyme inhibitor, and Ion channel modulator of 1.06, 0.75, and 0.28, respectively, shows that ABA may act as an active inhibitor for different insulin receptor proteins.

### 2.11. In Silico Pharmacokinetics and ADMET Evaluation of Abscisic Acid

**Figure 11:**
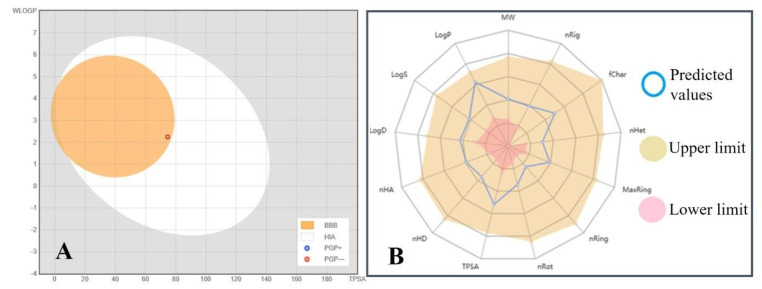
(**A**) A BOILED-Egg plot shows the blood-brain barrier (BBB) penetration and ABA molecules’ human intestinal absorption (HIA). Here PGP- shows the P-glycoprotein substrate negative nature of ABA. (**B**) Radar graph showing upper, lower, and predicted values of various physicochemical and molecular properties of ABA (Abbreviations: MW = Molecular weight, nRig = Number of rigid bonds, fChar = Formal charge, nHet = Number of heteroatoms, MaxRing = Number of atoms in the biggest ring, nRing = Number of rings, nRot = Number of rotatable bonds, TPSA = Toplogical polar surface area, nHD = Number of hydrogen bond donors, nHA = Number of hydrogen bond acceptors, LogP = Log of the octanol/water partition coefficient, LogS = Log of the aqueous solubility, LogD = Log at physiological pH 7.4.

The pharmacokinetic properties and drug-likeness data are summarized in Table 3. According to the pharmacokinetic/ADMET properties, ABA showed high (96.712%) human intestinal absorption (HIA) and very low BBB permeability (−0.047 log BB). On the other hand, ABA did not affect Cytochrome P450 isomers (CYP1A2 and CYP2D6). The drug-likeness prediction was also performed using the Lipinski, Ghose, and Veber rules, as well as the bioavailability score. The Lipinski (Pfizer) filter is the pioneer to filter out any drug at the absorption or permeation level that an ideal drug has a molecular weight of less than 500 g/mol, a log P value of less than 5, and a maximum of 5 H-donor and 10 H-acceptor atoms [40]. The drug-likeness requirements are defined as follows by the Ghose filter (Amgen): The computed log P ranges from −0.4 to 5.6, the MW ranges from 160 to 480, the molar refractivity (MR) ranges from 40 to 130, and the total number of atoms ranges from 20 to 70 [41]. Veber (GSK) rule defines drug-likeness constraints as Rotatable bond count ≤ 10 and topological polar surface area (TPSA) ≤ 140 [42]. According to Martin et al., the bioavailability score was implemented to predict the probability of a compound to have at least 10% oral bioavailability in rat or measurable Caco-2 permeability [43]. AMES toxicity (non-mutagenic), hepatotoxicity, or skin sensitization was not found in the ABA. By the overall analysis of Table 3, we conclude that ABA does not violate any existing drug-likeness rules like Lipinski, Ghose, Veber, Egan and Muegge. Meanwhile, ABA has physicochemical, molecular, and ADMET properties between the upper and lower predicted values (Table 3 and Figure 11A,B).

### 2.12. Boiled-Egg Plot and Radar Graph Analysis

A BOILED-Egg plot predicts the gastrointestinal absorption and brain penetration of small molecules. Here PGP+/− shows the P-glycoprotein substrate positive/negative nature of the molecule under study. The BOILED-Egg’s white (white area) predicts that the molecule located in this area may be passively absorbed by the human intestinal tract (HIA). The ABA molecule (red circle) is located at the extreme periphery of BOILED-Egg’s yolk (yellow area), which predicts that the ABA molecule may passively permeate through the blood-brain barrier (BBB) but have very low chances of −0.047 log BB, contrary to the chances of ABA being absorbed by the human intestinal tract (HIA) is 96.712% (Table 3 and Figure 11).

The radar graph shows that various physico-chemical and molecular characteristics of ABA like LogP (2.342), LogS (−2.465), LogD (1.656), molecular weight (264.32 g/mol), nHA (4), nHD (2), number of rotatable bonds (3), number of rings (1), rigid bonds (10), heteroatoms (4), and atoms in the biggest ring number of rings (6), formal charge (0), and topological polar surface area (74.60 Å²) have optimum values within the upper and lower limit (Table 3 and Figure 11).

## 3. Materials and Methods

### 3.1. Retrieval and Preparation of Proteins and Ligand

The human proteins related to diabetes mellitus 11-β-hydroxysteroid dehydrogenase (PDB ID-4K1L), aldose reductase (PDB ID-3G5E), glucokinase (PDB ID-4IXC), glycogen synthase kinase-3 (PDB ID-3F7Z), glutamine:fructose-6-phosphate amidotransferase [GFAT (PDB ID-2ZJ4)], pyruvate dehydrogenase kinase (PDB ID-4MP2), peroxisome proliferator-activated receptor-gamma (PDB ID-3DZY), sirtuin family of NAD(+)-dependent protein deacetylases [SIRT6 (PDB ID-3K35)], and Tyrosine kinase (PDB ID-1IR3) were retrieved from the RCSB Protein Data Bank (Rutgers University, NJ, USA) (Figure 1) [44]. The human pyruvate dehydrogenase kinase (PDB ID: 4MP2) protein molecule showed conformational error in 12 amino acid residues (VAL373, ARG370, VAL357, MET349, PHE347, VAL261, HIS247, HIS237, LEU211, GLN82, SER29, SER27), which were rectified by protein preparation and energy minimization wizard employing Discovery Studio version 2.0. The remaining PDB IDs did not show any structural error.

The ligand molecule ABA [(PubChem ID-5280896), IUPAC name- (2Z,4E)-5-[(1S)-1-hydroxy-2,6,6-trimethyl-4-oxocyclohex-2-en-1-yl]-3-methylpenta-2,4-dienoic acid)] in 2D (SDF) format was downloaded from PubChem (https://pubchem.ncbi.nlm.nih.gov/compound/5280896) (accessed on 15 April 2021) (Figure 1). The ligand molecule was converted into 3D format (.mol2 and .pdb) employing the Chem3D 16.0 module of ChemOffice 2016 software suit.

Before docking, the protein structures downloaded from PDB were analyzed in PyMol software (The PyMOL Molecular Graphics System, Version 2.0 Schrödinger, LLC, San Diego, CA, USA), and the already docked ligands or nucleic acid or heteroatoms or water molecules were removed from the X-ray crystallographic protein-ligand complexes. Then the pure proteins as a receptor were prepared in Swiss-Pdb viewer (version 4.1.0) by optimizing bonded atoms, angles, torsions, non-bonded atoms, and improper atoms of the protein backbone and side chains.

### 3.2. Molecular Docking

The docking calculations were performed using the AutoDock Vina version 4.2 (ADT4.2) software suite [45]. The receptor proteins were solvated with water, and only polar hydrogens were added. The receptor grid boxes (in X, Y, Z dimension) were prepared in the ADT4.2, and the pdbqt files of proteins were generated. Similarly, the ligand was prepared with default parameters, and only Gasteiger charges were added. Flexible Ligand docking was performed applying the Lamarckian Genetic Algorithm with an exhaustiveness value of eight. The contributions of intramolecular hydrogen bonds, hydrophobic, ionic, and Van der Waals interactions between docked protein and ligand complexes were used to determine the free energy (ΔG) specifying affinity scoring of the binding. The docking poses were narrowed down using the force field’s free binding energy computation. After the docked protein-ligand complexes were created, the binding sites were analyzed to construct a 2D representation of the ligand interaction for each complex.

### 3.3. Post-Docking Protein-Ligand Interaction Analysis

The visualization and analysis of protein-ligand complexes were performed using PyMOL software (The PyMOL Molecular Graphics System, Version 2.0 Schrödinger, LLC, San Diego, CA, USA). The receptor’s active sites and interactions with the ligand or drug were determined using the PDBe [46] and PDBsum [47] servers. The protein-ligand complexes were further visualized in the Discovery Studio client v21.1.0.20298, Dassault Systemes Biovia Corp, to show the 2D diagram of ligand-receptor interaction. LigPlot+ and Maestro12.4 (Schrodinger-2020-2) were applied to visualize ligands’ exact atomic level interaction with their corresponding receptor atoms.

### 3.4. Calculation of Inhibition Constant

Moreover, it is concluded that ABA may act as a competitive inhibitor that should compete with the known substrates to the active centers of the protein targets relevant to diabetes mellitus. Therefore, it induces a competitive type of inhibition, that inhibitors could bind to only the free enzyme and formed reversible enzyme-inhibitor (E-I) complexes. An enzyme-inhibitor complex’s inhibition constant (Ki) is traditionally calculated by the basic equations of enzyme kinetics of the Lineweaver–Burk assay extrapolated on 2D plots. If there is inconsistency in the Lineweaver–Burk plots, non-linear regression of the Michaelis–Menten equation is used to validate the related constants obtained.

Sophisticated arithmetic and analytical in silico algorithms have been proposed to compute the inhibition constant (Ki) parameter, since the Ki principally depends on the binding (or association) constant (Kb) and dissociation constant (Kd) of an enzyme-inhibitor complex, which occurs in opposite directions (ln Kb = −ln Kd).

ΔG = (R × T) lnKi

Therefore, Ki is computationally calculated using the following formula:

Ki = exp(ΔG/(R × T))

The binding energy ΔG is in kcal/mol, the universal gas constant R = 1.987 kcal/K/mol, at room temperature (25 °C) T = 273 + 25 = 298 K. Ki is having a unit of mM.

### 3.5. Screening of Ligand Abscisic Acid for Pharmacodynamics Properties

The molinspiration (https://www.molinspiration.com/cgi-bin/properties), (accessed on 29 May 2021), an online screening server based on sophisticated Bayesian statistics, was implemented to analyze the pharmacodynamics properties of ABA. It compares representative ligands’ structures and determines physico-chemical properties of a particular molecule for being active molecules. There is no need to know about the target’s 3D structure or binding mode. The trained model makes it possible to screen large libraries of hundreds of thousands of molecules in less than an hour to identify molecules with the highest chance of becoming active drugs, pesticides, irritants, or toxic substances. The larger the value of the bioactivity score is, the higher the probability that the particular molecule will be active.

### 3.6. Screening of Ligand Abscisic Acid for Pharmacokinetics and Drug-Likeness

The main causes of drug development failure are undesirable pharmacokinetics and toxicity of candidate molecules. Absorption, distribution, metabolism, excretion, and toxicity (ADMET) of chemicals have long been recognized as important considerations in the early stages of CADD.

The pkCSM [48], SwissADME [49] and ADMETLAB2.0 [50] are free web tools to evaluate Pharmacokinetics, drug-likeness, and medicinal chemistry of small molecules based on very extensive experimental data sets. The SMILES format of the molecule was entered, and 2D structure files were generated in SwissADME, pkCSM, and ADMETLAB2.0. The pkCSM is an authentic source (collaboratively developed by Instituto Rene Rachou Fiocruz Minas, The University of Melbourne and University of Cambridge) to predict small-molecule pharmacokinetics using graph-based signatures. Several parameters are analyzed to check the ADMET properties of a small molecule or inhibitor.

SwissADME [51] of Swiss Institute of Bioinformatics used to evaluate the pharmacokinetics and drug-likeness ADMET behaviors of compounds [52] employing support vector machine (SVM) algorithm [53] with well-characterized large datasets of known inhibitors/non-inhibitors as well as substrates/non-substrates.

ADMETlab 2.0 has a greater capacity to assist medicinal chemists in accelerating the drug research and development process. It allows users to calculate and predict 17 physicochemical parameters, 13 medicinal chemistry measures, 23 ADMET endpoints, 27 toxicity endpoints, and eight toxicophore rules (751 substructures) quickly and easily, allowing them to identify interesting lead compounds for further investigation.

The major target of the study was to examine if the substance in question inhibited the cytochrome P450 (CYP) family’s CYP1A2 and CYP2D6 isoforms. Pharmacokinetics parameters such as human intestinal absorption, P-glycoprotein, and the BBB and drug-likeness prediction Lipinski, Ghose, and Veber criteria, as well as the bioavailability score, are crucial in judging the molecule [41,42,54]. According to several essential criteria such as molecular weight, LogP, number of HPA, and HBD, the Lipinski, Ghose, and Veber guidelines were used to assess drug-likeness to determine whether a compound is likely to be bioactive.

According to Lipinski’s “Rule of 5” most “drug-like” compounds have logP ≤ 5, molecular weight (MW) ≤ 500, number of hydrogen bond acceptors (nHA) ≤ 10, and number of hydrogen bond donors (nHD) ≤ 5 [55]. The molecule that violates more than one of these principles may have problems with bioavailability. The methodology calculates logP (octanol/water partition coefficient) as a sum of fragment-based contributions and correction factors. This approach is quite reliable, and it may be used to analyze almost any organic or organometallic compound.

Topological Polar Surface Area (TPSA) is calculated based on the methodology published by Ertl et al. (2000) depending on the fragment contributions [56]. TPSA is defined as the sum of surfaces of polar atoms (typically oxygens, nitrogens, and linked hydrogens) in a molecule. TPSA is an ideal descriptor characterizing drug absorption, including human intestinal absorption, bioavailability, Caco-2 (human epithelial colorectal adenocarcinoma cell line), monolayers permeability, and BBB penetration. These parameters are quite important in predicting drug transport qualities. The number of rotatable bonds (nRot) is a simple topological parameter that measures molecular flexibility. It is a very good descriptor of the oral bioavailability of drugs [42]. Any single non-ring bond bounded to a non-terminal heavy (i.e., non-hydrogen) atom is termed a rotatable bond. Because of their large rotational energy barrier, amide C-N bonds are not considered.

## 4. Conclusions

Effective medications with no cytotoxicity are needed to treat diabetes mellitus, and phytohormones such as ABA are among the best natural extract with no side effects. Molecular docking investigation of ABA with nine different protein targets relevant to diabetes mellitus revealed four potential target proteins perfectly docks with ABA. Docking analysis also revealed that based on binding energy (ΔG) and predicted inhibition constant (pKi), 11β-HSD1 (4K1L) showed best binding with ABA, followed by GFAT (2ZJ4), PPAR-gamma (3DZY), and SIRT6 (3K35), which were equal in inhibition constant. The docking and interaction pattern of ligands were fantastically able to interact with the key residues of the catalytic cavity of the enzyme or located in the very close proximity of the active sites of these proteins. Following all current drug-likeness guidelines such as Lipinski, Ghose, Veber, Egan, and Muegge, the pharmacodynamic and pharmacokinetic features with ADMET study revealed that ABA could be taken best molecule without any hazardous effect. A BOILED-Egg plot and radar graph analysis confirm that all the molecular and physico-chemical properties of ABA are within the upper and lower limit fulfilling all the criteria of an ideal drug. Thus, ABA can be considered a potential candidate for developing a potent anti-diabetic drug and a promising bioactive compound of okra for developing nutraceuticals and functional foods.

## Figures and Tables

**Table 1:** AutoDock Vina results showing binding energies and inhibition constant of ABA with different proteins related to diabetes mellitus.

S. No	Protein Name (PDB ID)	Theoretical Weight (KDa)	Name of Chains	Binding Energy (ΔG) (kcal/mol)	Predicted Inhibition Constant pKi (µM)	No. of H-Bonds	H-Bond Forming Residues
1	11β-HSD1 (4K1L)	31.84	A, B, C, D	−8.1	6.01	2	TYR(A)183, SER(A)169
2.	GFAT (2ZJ4)	42.32	A	−7.3	5.21	5	CYS373, THR375, GLN421, SER422, THR425
3.	PPAR-gamma (3DZY)	51.53	A, B, C, D	−7.3	5.21	2	TYR(A)189, TYR(D)250
4.	SIRT6 (3K35)	35.15	A, B, C, D, E, F	−7.3	5.21	2	GLN(C)111, HIS(C)131
5.	Glucokinase (4IXC)	50.81	A	−6.8	5.05	3	ASN83, ARG85, GLY229
6.	Aldose reductase (3G5E)	36.18	A	−6.6	4.84	2	TRP111, CYS298
7.	Glycogen synthase kinase-3 (3F7Z)	39.88	A, B	−6.6	4.84	0	-
8.	Pyruvate dehydrogenase kinase (4MP2)	45.23	A	−6.3	4.55	1	AGR162
9.	Tyrosine kinase (1IR3)	35.03	A	−6.2	4.47	1	GLU1043

**Table 2:** Prediction of bioactivity of ABA as an inhibitor against different insulin receptor proteins by molinspiration (v2018.03).

S. No	Parameters	Bioactivity Score
1	GPCR ligand	−0.01 ↓↓↓
2	Ion channel modulator	0.28 ↑
3	Kinase inhibitor	−0.61 ↓
4	Nuclear receptor ligand	1.06 ↑↑↑
5	Protease inhibitor	−0.20 ↓↓
6	Enzyme inhibitor	0.75 ↑↑

The upward arrow represents high bioactivity while downward arrow is showing low bioactivity of ABA.

**Table 3:** In silico pharmacokinetics, physico-chemical, ADMET properties, and drug-likeness of ABA.

Physicochemical Properties	Predicted Values	Absorption	Predicted Value	Distribution	Predicted Values	Metabolism	Predicted Value	Extraction and Toxicity	Predicted Value
LogP, LogS and LogD	2.342, −2.465 and 1.656	Water solubility logP	−2.253 mol/L	Volume distribution (VD) of a drug in blood plasmas	0.343 L/kg	CYP2D6 substrate and CYP3A4 substrate	No	Total drug clearance log (CLtot)	0.685 mL/min/kg
Molecular weight	264.32 g/mol	Lipd solibility LogP	1.96 Log Po/w	Plasma protein binding (PPB)	79.896%	CYP2D6 inhibitor	No	Renal organic cation transporter (OCT2) substrate	No
Number of hydrogen bond acceptors (nHA), donors (nHD), and rotatable bonds (nRot)	4, 2 and 3	Caco2 permeability	0.913 og Papp in 10–6 cm/s	The fraction unbound in blood plasmas (Fu)	9.191%	CYP3A4 inhibitor	No	AMES toxicity, hepatotoxicity, skin sensitization, hERG I & II inhibitor	No
Number of rings (nRing), rigid bonds (nRig), heteroatoms (nHet), and atoms in the biggest ring (MaxRing)	1, 10, 4 and 6	Log Kp skin permeability	−2.715 cm/s	BBB permeability	−0.047 log BB	CYP1A2 inhibitor	No	Max. tolerated dose (human)	0.304 log mg/kg/day
Formal charge (fChar)	0	Human intestinal absorption (HIA)	96.712%	CNS permeability	−2.913 log PS	CYP2C19 inhibitor	No	Oral rat acute toxicity (LD50)	1.793 mol/kg
Molecular total polar surface area (TPSA)	74.60 Å²	P-glycoprotein substrate, P-glycoprotein I & II inhibitor	No	Bioavailability score	0.85	CYP2C9 inhibitor	No	*T. Pyriformis* toxicity	0.42 log ug/L

## Data Availability

All data generated or analyzed during this study are included in this article.

## Sample Availability

Samples of the compounds are not available from the authors.
